# Genetics of multiple endocrine neoplasia type 1 syndrome: what's new and what's old

**DOI:** 10.12688/f1000research.7230.1

**Published:** 2017-01-24

**Authors:** Alberto Falchetti

**Affiliations:** 1 EndOsMet Unit, Villa Donatello, Piazzale Donatello 2, Florence 50100, Italy; 2 Hercolani Clinical Center, Via D’Azeglio 46, Bologna 40136, Italy

**Keywords:** Multiple Endocrine Neoplasia Type 1 Syndrome, Menin, familial MEN1, genetic screening MEN1, MEN1 gene mutation

## Abstract

Despite its identification in 1997, the functions of the *MEN1* gene—the main gene underlying multiple endocrine neoplasia type 1 syndrome—are not yet fully understood. In addition, unlike the *RET*—MEN2 causative gene—no hot-spot mutational areas or genotype–phenotype correlations have been identified. More than 1,300 *MEN1* gene mutations have been reported and are mostly "private” (family specific). Even when mutations are shared at an intra- or inter-familial level, the spectrum of clinical presentation is highly variable, even in identical twins. Despite these inherent limitations for genetic counseling, identifying *MEN1* mutations in individual carriers offers them the opportunity to have lifelong clinical surveillance schemes aimed at revealing MEN1-associated tumors and lesions, dictates the timing and scope of surgical procedures, and facilitates specific mutation analysis of relatives to define presymptomatic carriers.

## Introduction

Multiple endocrine neoplasia type 1 syndrome (MEN1, MIM*131100) is an autosomal dominant disorder in which varying combinations of either endocrine or non-endocrine tumors may present extremely varied phenotypic clinical patterns. Considerable phenotypic variability of tumor type manifestations and age at diagnosis has been reported, even within the same family, whose affected members share the same, inherited, *MEN1* gene mutation ^1^.

The original description of the classical “P-triad” corresponds to parathyroid, pituitary, and pancreatic (neuro)endocrine tumors ^2^. Other MEN1-associated endocrine (adrenocortical tumors and carcinoids) and non-endocrine, mostly benign, neoplasms (facial angiofibromas, collagenomas, and others) may also occur ^3, 4^, and other types of tumors (e.g. adrenal) are occasionally reported in the literature. Despite the frequent occurrence of endocrine neoplasm combinations, findings so far suggest that a genetically predisposed abnormal proliferative control may exist in practically all of the mutant cells of a MEN1-affected individual.

Much of the well-established knowledge on MEN1 has been already described in my previous F1000 Faculty review ^5^; however, information on new aspects is lacking. In this short review, attention to the role of genetics in the clinical management of MEN1-affected subjects will be presented, including also some new technical and practical aspects.

## Mendelian genetics of MEN1 syndrome

The estimated worldwide prevalence of MEN1 is expected to be between 1 in 30,000 and 1 in 500,000 ^3, 6^, but some geographical clustering due to founder effects has also been reported ^7, 8^. MEN1 syndrome may occur in either familial or sporadic forms. The sporadic form of MEN1, where only one affected person is identified in a previously unaffected family, is observed much less frequently (10% of cases) than the familial form (90% of cases).

MEN1 is clinically defined when at least two first-degree relatives have a combination of either one of the three main endocrine tumors. Alternatively, it involves only one of the main target organs and a *MEN1* disease-causing germline mutation. As MEN1 syndrome follows an autosomal dominant inheritance pattern, the offspring of an affected mutation carrier has a 50% chance of inheriting the pathogenic mutation ^9^. As already mentioned, it is not only members of the same family who may have diverse clinical features but also MEN1 monozygotic twins who have been reported to exhibit differing symptoms ^10^. However, the distinction between sporadic and familial cases is not always easy. In some sporadic cases, where family history cannot be ascertained, this may be attributed to non-paternity, early parental death, lack of careful family assessment, and adoption ^11^.

MEN1 penetrance is high, with more than 95% of *MEN1* mutation carriers having biochemical evidence of MEN1—generally represented by mono- or pluri-hormones over secretion—with 100% presenting with hyperparathyroidism by 50 years of age and approximately 80% of patients presenting clinical signs by the fifth decade of life ^12^ ( Table 1). In fact, patients with MEN1-related primary hyperparathyroidism (PHPT) exhibit a higher susceptibility to nephrolithiasis than do non-MEN1-PHPT patients ^13– 15^, as there is also a higher frequency of renal calculi in MEN1 patients before 30 years of age ^16^.

**Table 1.** Age-related penetrance by 50 years of age for “classical” MEN1-associated tumors ^60, 63^.

MEN1-associated endocrine disorder	Age-related penetrance by 50 years of age
Primary hyperparathyroidism (multiglandular disease)	73–75%
Pituitary adenomas	31–48%
Islet cell tumors*	45–49%

*This classification considers only the “old, classical” functioning pancreatic endocrine tumors, as originally described in the literature, but currently with the widespread use of endoscopic ultrasound in the work up of MEN1, duodenal-pancreatic neuroendocrine tumors, mostly nonfunctioning, are found in more than 80% of patients and their early occurrence has also been demonstrated ^13– 15^. However, the age of presentation of specific tumor types is highly variable, ranging from 9–25 years of age for the youngest diagnosed case to 68–77 years for the oldest case with a tumor manifestation ^60^.

## The *MEN1* gene and its encoded product, menin

The *MEN1* gene localizes to chromosome 11q13 ^17– 19^ and consists of 10 exons encoding a 610-amino-acid protein called menin. Menin is ubiquitously expressed and is predominantly located in the nucleus in non-dividing cells ^20– 24^.

Menin is extremely functionally versatile. It shows no homology with other known proteins and the mechanism by which its loss of function leads to MEN1 is still unclear ^20– 22^. Menin primarily localizes to the nucleus; it contains two classical nuclear localization signals (NLSs) and at least one further non-classical NLS in its C-terminus ^23, 24^. At the nuclear level, menin can associate with chromatin ^25^, double-stranded DNA ^26^, the lysine-specific histone methyltransferases KMT2A and KMT2B ^27, 28^, and components of a transcriptional repressor complex, including histone deacetylases (HDACs) ^29^.

Menin interacts with transcription factors, such as activating protein-1 (AP-1), JunD, nuclear factor-κB (NF-κB), β-catenin, mothers against decapentaplegic (SMAD) family members, and estrogen receptor α (ERα) ^27, 30– 36^. It is also able to bind to cytoskeletal proteins, such as vimentin ^37^, and cytoplasmic cell signaling mediators, including Akt1/protein kinase B (PKB) and Forkhead box protein O1 (FoxO1) ^38, 39^. In addition, it has been shown that menin plays a role in cell proliferation ^40– 42^, apoptosis ^43, 44^, and genome integrity ^45^.

The menin/KMT2A complex also regulates the expression of several Hox genes as well as *CDKN1B*, a gene that harbors inactivating mutations accounting for multiple endocrine neoplasia type 4 (MEN4) syndrome (MIM #610755). The same protein complex interacts with ERα and co-activates ERα-mediated transcription ^27, 36, 46, 47^. KMT2A, located at 11q23.3 chromosome, harboring recurrent chromosomal breakpoints, is disrupted in distinct 11q23 recurrent chromosomal translocations ^48^. Its rearrangement alleles encode mixed-lineage leukemia (MLL) fusion proteins (MLL-FPs) or internal gene rearrangement products. Interestingly, chromosomal rearrangements involving KMT2A lead to MLL, and, in this context, menin was shown to be required for KMT2A-dependent oncogenic transformation ^49^. Some authors have produced robust data showing that combining two targeted treatments, DOT1L (H3K79 methyltransferase) and menin inhibition, may result in a promising therapeutic strategy for MLL-rearranged leukemia ^50, 51^.

The functional versatility of menin in different tissues may be key in unraveling the as-yet-unexplained tissue selectivity of MEN1-associated tumors as well as the variable phenotypic expression of an identical mutant allele.

## Genetic testing and screening in MEN1

Although MEN1 is a rare disorder, the autosomal dominant inheritance form implies that detecting a germline *MEN1* mutation in a single familial member has important implications for other family members. In particular, first-degree relatives have a 50% risk of possessing the familial mutation with the consequent high risk for developing MEN1-associated tumors ^1^. Thus, screening for MEN1 involves both clinical and imaging detection of associated tumors and ascertainment of their germline genetic state: normal or mutant gene carrier ^52^. Cloning of the *MEN1* gene ^20^ facilitated the identification of asymptomatic mutation carriers, who are genotypically assigned a high-risk status for developing MEN1-associated tumors and are offered an early detection scheme ^5^.

Moreover, a potentially “new” MEN1 family could also be identified when it first appears in a subject lacking a clear familial history. Thus, affected relatives have the opportunity to be included in a specific surveillance schedule and receive therapy as soon as possible ^53^.

## Linkage analysis approach: it cannot be considered totally obsolete

In the past, before cloning the *MEN1* gene in 1997 ^20, 21^, linkage analysis was the only clinically useful approach for genetic diagnosis; it uses highly polymorphic DNA markers located upstream and downstream of 11q13, the chromosomal region to which the *MEN1* gene was mapped ^17– 19^. Since some of these DNA markers show no recombination with the *MEN1* gene (i.e. PYGM, D11S463, and D11S427), an accuracy of up to 99.5% could be reached in the test for carriers, with incorrect results due to meiotic crossing over being omitted ^5, 21^. However, for such analysis, there needs to be a MEN1 family with two or more living, clinically affected members, bridging two or more generations, allowing for the detection of the family-specific 11q13 risk haplotype in affected people ^53^.

One obvious limitation is genetic heterogeneity with an overlap between MEN1 and MEN4. Another important limitation of this screening technology is that it cannot be applied to a single index case. However, the linkage approach should be considered when mutational analysis fails to detect any germline *MEN1* mutation in a proband and the pedigree is informative (more affected members from different generations).

Finally, it has been also reported that applying forensic techniques to analyze ancient DNA enables the identification of the familial disease-associated haplotype, demonstrating that even when one or more relatives are no longer living, the family history relating to MEN1 can still be assembled ^54^.

## Mutations of the *MEN1* gene

In the 10 years following the identification of the MEN1 gene, a total of more than 1,300 mutations (approximately 85% germline and 15% somatic) were characterized ^55^, with the current total number of mutations at over 1,800 ( http://www.umd.be/MEN1/ ^56^). The germline *MEN1* mutations consist of 459 different mutations, which are distributed throughout the whole 1830 bp coding region and splice sites of the *MEN1* gene ^20, 21, 57– 59^ ( Table 2).

**Table 2.** Different types of *MEN1* gene mutations reported in the literature and their frequencies ^57^.

Types of *MEN1* gene mutations	Percentage
Nonsense	23%
Frameshift deletions or insertions	41%
In-frame deletions or insertions	6%
Splice site	9%
Missense	20%
Whole gene or particular gene deletions	1%

However, around 5–10% of MEN1-affected individuals may not harbor mutations in the *MEN1* gene coding region ^20, 21, 57– 60^; they may have whole gene deletions or mutations in the promoter or untranslated regions that have not been reported to date. Large deletions will not be easy to detect by conventional Sanger sequencing, but next-generation sequencing (NGS) technology enables us to extrapolate the large gene rearrangements. No studies that have employed these novel techniques in *MEN1* gene analysis have been published yet.

Approximately 75% of *MEN1* mutations are inactivating ^55^, as expected for a tumor suppressor gene. There are many different types of mutations, and they are dispersed throughout the coding region of the gene rather than being clustered, as predicted from pathogenic mutations in a tumor suppressor gene. A few of the mutations have occurred a number of times in unrelated families, and mutations at nine sites in the *MEN1* gene account for over 20% of all of the germline mutations ( Table 3).

**Table 3.** The nine recurring mutations by type ^55, 56^.

Type of *MEN1* mutations	Localization within the gene (there is more than one mutation in most of each of the following codons)
Deletions or insertions	Codon 83
	Codon 84
	Codon 120
	Codons 210–211
	Codons 514–516
Novel acceptor site	Intron 4
Nonsense	Arg98Stop
	Arg415Stop
	Arg460Stop

These recurring mutations could signify possible “hot spots”, and the deletional and insertional hot spots may correlate with DNA sequence repeats, DNA stretches of long strips of either single nucleotides or shorter repeat elements, ranging from dinucleotides to octanucleotides ^59^. Thus, a replication-slippage model could be in place at different codons, meaning that the *MEN1* gene seems to include DNA sequences that may make it prone to deletional and insertional mutations ^59^. There is no evidence that the promoter region of the *MEN1* gene contains any mutations, but this intriguing, albeit theoretical, possibility remains ^61^.

## What to do when a *MEN1* gene mutation is not detected at DNA sequencing: alternative approaches

In a relatively small percentage of patients with MEN1 (5–10%), gene mutations in either the coding region or the splice sites of the *MEN1* gene are not identified ^55^. Consequently, it cannot be excluded that pathogenic sequence variants in the promoter, deep in the introns, or in the untranslated regions (gene regions normally not analyzed in “routine” genetic tests) may exist. In addition, gross deletion/insertion of parts of the gene or even the entire gene cannot be detected with classical *MEN1* Sanger sequencing analysis.

Southern blot analysis or other gene dosage procedures (e.g. array comparative genomic hybridization [CGH]) or NGS could be useful to detect “gross” alterations at the *MEN1* gene, such as large deletions, insertions, or other large genomic rearrangements involving the *MEN1* gene. Multiplex ligation-dependent probe amplification (MLPA) is a quantitative and very sensitive and accurate multiplex polymerase chain reaction-based approach that enables the detection of copy number changes within a specific gene. Therefore, it is also possible to reveal whole gene and/or entire exon losses as gross modifications at the intra-genic level. Diagnostic screening by MLPA should be considered in MEN1 index cases in which we have a negative sequencing *MEN1* gene test result and large deletions/duplications of the *MEN1*-coding region which need to be assessed/excluded. As mentioned above, familial haplotype analysis should still be considered when either sequencing or MPLA screenings are negative ^62^.

## Attention to *MEN1* gene polymorphisms

Since 24 polymorphisms (12 in the coding region [10 synonymous and two non-synonymous], nine in the introns, and three in the untranslated regions) of the *MEN1* gene have been described ^55^, it is important to consider their occurrence, as they need to be differentiated from mutations when mutational analysis for genetic diagnosis is performed.

## *MEN1* phenocopies

Finally, since less than 2% of clinical MEN1 patients lack evidence of *MEN1* mutation, in cases where patients present with classic MEN1 symptoms but negative results for *MEN1* and *CDKN1B* mutations, further investigation of genes encoding members of the cyclin-dependent kinase inhibitor (CDKN) family—such as CDKN1A (p21cip1), CDKN2B (p15Ink4b), or CDKN2C (p15Ink4c), which all negatively regulate cell cycle progression and cell growth ^55^—should be considered ^53^.

## Genotype–phenotype correlations

There is no correlation between *MEN1* mutation location along the gene or the type of mutation and clinical manifestations. This lack of genotype–phenotype correlation, in addition to the sheer number of possible mutations in the coding region of the *MEN1* gene, results in greater difficulty for mutational analysis in the diagnosis of MEN1 than in the diagnosis of MEN2 ^10^.

One noteworthy study found that all patients with *MEN1* frameshift mutations have PNETs ^63^, while another showed a higher rate of malignant tumors for mutations in *MEN1* gene exons 2, 9, and 10 ^64^. However, no genotype–phenotype correlation could be consistently confirmed in other patient populations by other investigators ^12, 55^. Moreover, studies of unrelated kindreds exhibiting the same *MEN1* mutation showed large variability of different associated tumors ^11, 58^—as mentioned above, there are reports of identical twins who carry an identical *MEN1* mutation with different MEN1 clinical phenotypes ^10, 65, 66^. Finally, whereas some families with particular *MEN1* mutations develop only isolated hyperparathyroidism, other families with the same mutations develop a full MEN1 spectrum ^55^.

## Has the mutational analysis of the *MEN1* gene improved the life expectancy associated with the syndrome?

Although MEN1 patients have been reported to exhibit a decreased life expectancy, MEN1-associated mortality ( Table 4), mostly due to gastroenteropancreatic malignancy ^12, 67– 70^, has improved since the 1980s owing to both early detection of asymptomatic/presymptomatic *MEN1* mutation carriers and more intense clinical screening programs, with an overall better perioperative survival, especially for neuroendocrine tumors (NETs) of the gastrointestinal tract, together with appropriate drug treatment, when applicable ^69^.

**Table 4.** Cause of death due to MEN1-associated malignancies. Patients affected by these malignancies have a threefold higher risk of death ^69^.

MEN1 tumors with high risk of death	Percentage
Malignant neuroendocrine gastroenteropancreatic tumors (mainly gastrinomas) Thymic or bronchial carcinoid tumors	30–40%

Thus, the early genetic diagnosis of *MEN1* is strictly recommended in order to both identify patients before biochemical/clinical manifestations occur and improve long-term outcome. Periodical screening and clinical follow-up according to clinical guidelines have to be performed for all MEN1 patients in order to offer appropriate and early medical/surgical interventions ^71, 72^, and, more in general, it has also been suggested that early genetic screening for those syndromes in which NETs may occur as a hereditary feature may contribute to related morbidity/mortality reduction in asymptomatic subjects through better and more appropriate clinical management ^73^.

It seems that we can offer MEN1 patients a better prognosis and a reduction in morbidity and mortality if early stage diagnosis of the tumor is achieved, along with presymptomatic tumor detection and early administration of specific therapy ^13– 15^.

## MEN1 tumorigenesis: not only loss of heterozygosity

Consistent with Knudson’s two-hit hypothesis ^17, 18, 74^, the *MEN1* gene is thought to act as a tumor suppressor, since over 90% of tumors from MEN1 patients exhibit loss of heterozygosity (LOH). However, intra-genic deletions and point mutations can also be responsible for inactivating the wild-type allele—the second hit. In fact, some MEN1 tumors where no LOH was demonstrated have been shown to harbor different somatic and germline-inactivating point mutations of the *MEN1* gene ^75^, mechanisms still consistent with the Knudson two-hit hypothesis ^76^.

## Any role for epigenetic and/or modifying genetic mechanisms in the clinical expression of *MEN1*?

Since it is known that menin is an essential component of histone methyltransferase complexes that contain members from the MLL and trithorax protein family ^27^, by acting as a scaffold protein, it may epigenetically regulate gene expression via histone methylation or acetylation. Consequently, it has also been suggested that epigenetic mechanisms triggered by environmental factors may influence the disease phenotype in patients carrying the same *MEN1* mutation ^77^. Moreover, as recently reported, a specific variant of the *CDKN1B* gene whose inactivating mutations account for the MEN4 syndrome was demonstrated to be disease modifying in MEN1 patients with truncating *MEN1* mutations, causing a higher number of MEN1-related tumors ^78^.

## Could microRNA molecules play a role in MEN1 tumorigenesis? The miR-24 experience

It has been described that a microRNA molecule, miR-24-1, is able to bind to the 3′ untranslated region of *MEN1* mRNA. miR-24-1 expression profiles have been conducted in some MEN1 parathyroid adenomas from *MEN1* mutant carriers, their sporadic non-MEN1 counterparts, and in normal parathyroid tissue. The results suggest that MEN1 tumorigenesis may be under “negative feedback loop” control between miR-24-1 and menin, thus mimicking the Knudson’s second hit and possibly buffering the effect of the stochastic factors hypothesized to contribute to the onset and progression of MEN1 disease ^79^. If such findings are confirmed by other studies in other MEN1 tumors from subjects with the same or different *MEN1* gene mutations, they could suggest the existence of an alternative pathway to MEN1 tumorigenesis and, probably, to the ‘Knudson’s two-hits dogma or, maybe, an alternative MEN1 tumorigenesis for specific MEN1-affected endocrine and non-endocrine tissues.

Overall, this could be considered as a new basis for future developments in RNA antagomir(s)-based strategies to control tumorigenesis in *MEN1* carriers.

## Could variants in genes other than *MEN1* be disease modifying?

Recently, other genetic mechanisms have been investigated for their possible involvement in MEN1-related pleiotropic phenotypic expression. It is known that the p27 ^Kip1^ protein, encoded by the *CDNK1B* gene, is downstream of MEN1-driven tumorigenesis. Genotypic frequencies of the *V109G* variant of p27 have been evaluated in a cohort of MEN1 patients and healthy controls and *V109G* seems to influence the clinical manifestation of adult MEN1 patients carrying truncating *MEN1* gene mutations ^78^.

Since menin forms a transcriptional complex with MLL2 and RNA polymerase II, regulating p27 ^Kip1^ expression, inactivation of menin reduces p27-mRNA levels and a second hit event, as the occurrence of *V109G* variant, potentially correlated with p27 protein degradation by p38JAB1, a protein promoting the degradation of this cyclin-dependent kinase inhibitor, may trigger exaggerated multiple tumor developments.

More recently, it has been suggested that such a polymorphism may be associated with certain *MEN1* mutations ( *c.502G>A*, *p.G168R* in exon 3, *c.673T>A, p.W225R* in exon 4, and *c.825 + 1G>A* in intron 5), and carriers of both the genetic variants, *MEN1* mutation and *V109G*, seem to exhibit a more aggressive clinical course of the syndrome with a worse prognosis ^80^.

All of the above reported findings need to be replicated in other, ethnically diverse MEN1 clinical series.

## Future perspectives in MEN1 genetic analysis

Recent developments have seen a new era for sequencing in several Mendelian diseases in the form of NGS technology, which could be helpful for bypassing the limitations of “classical” genetic analysis, as described above. The NGS approach as a genetic diagnostic tool could permit simultaneous sequencing of the following extra-/intra-genic regions: a) regulating and untranslated, b) coding sequences, and c) introns. Thus, such an approach may allow the identification of either causative large intra-genic deletions/duplications or novel mutations ^81^. Specifically, clinically relevant chromosomal rearrangements, such as the ones occurring at KMT2A (MLL), can be detected by targeted gene panel-based NGS that has a sensitivity and specificity equivalent to fluorescence *in situ* hybridization protocols, and reverse transcription polymerase chain reaction approaches, as well as more detailed information and better efficiency for molecular testing. Furthermore, translocation detection by NGS offers more advantages than the “conventional” laboratory methods, such as the more precise definition of the breakpoint region and the detection of both cryptic rearrangements and unknown molecular partner genes while it runs parallel with gene mutation detection ^82^. Moreover, NGS could extend the sequencing of nucleotides from a single gene up to the multigene level by specifically setting up targeted panels, up to the whole genome, producing huge amounts of genetic data on a gigabyte scale in a single step. NGS may represent a higher-throughput alternative to classical DNA sequencing as well as being less expensive when compared to the traditional method.

In addition, NGS is very flexible, reaching an adequate resolution level for any single genetic analysis, also considering that a sequencing run can be specifically tailored to obtain genetic data and/or to screen one or more predetermined genomic regions or a specifically desired gene set.

Laboratory protocols such as whole-exome sequencing (WES) and whole-genome sequencing (WGS) enable us to analyze an untargeted exome- or genome-wide section of an individual’s DNA and, at least theoretically, detect every genetic variant in a subject. Such approaches in parent–offspring trio models may be helpful in determining inherited variants as well as *de novo* mutations in the offspring and will enhance our ability to identify most disease-causing genes contributing to sporadic monogenic disorders, such as MEN1 syndrome ^83^.

In summary, overall, two alternative protocols are currently available to detect gene mutations: (1) WES and WGS, facilitating the identification of disease-associated genes and/or regulatory elements, even those not previously known––this approach tends to be useful for genetically determined diseases whose responsible gene/genes are still unknown; and (2) NGS-targeted multi-gene sequencing by selecting a platform with specific genes comprising coding, non-coding, and regulatory gene regions.

Implementation of all of these procedures will facilitate, in the next few years, the genetic diagnosis of diseases or groups of related disorders, such as multiple endocrine neoplasia syndromes, making their differential genetic diagnosis possible by creating an up-to-date specific platform that includes all specifically relevant genes ^84, 85^. In such a way, it will soon be possible to classify different human oncological disorders, including MEN1 syndrome, according to the underlying genotype rather than solely the biochemical/clinical phenotype. Thus, in the near future, it will be possible for MEN1-affected subjects to have targeted medical consultations and interventions and engagement in gene-specific patient groups, as well as more appropriate treatments ^83^.

## Current limitations and advantages in MEN1 genetic diagnosis

### Limitations

***Lack of genotype/phenotype correlation.*** As stressed here and in my previous F1000 Faculty review, we do not have any genotype–phenotype correlation. Thus, whether a specific *MEN1* mutation is detected and/or it localizes to a specific functional domain of menin still does not improve specific clinical predictions of disease occurrence, symptoms, or progression. Consequently, genetic information currently has limited importance in the individual clinical management of mutation carriers whether or not they are displaying symptoms.

### Advantages

***Identification of germline *MEN1* gene mutation.*** Clearly, the identification of a pathogenic *MEN1* mutation is useful for ensuring an individual’s inclusion in clinical surveillance routines for MEN1-associated tumors and lesions ^5^, suggesting specific surgical procedures, and identifying the need for specific mutation analysis of first-degree relatives to identify presymptomatic mutation carriers. In the presence of a germline *MEN1* mutation, lifelong specific clinical surveillance is suggested, as reported in the literature ^86^.
